# Evaluating the role of renewable energy natural resources and globalization in environmental quality in OIC countries

**DOI:** 10.1038/s41598-025-16872-y

**Published:** 2025-09-29

**Authors:** Azizullah Faizi, Mehmet Zeki AK, Talal H. Alsabhan, Mohammad Rahim Shahzad, Reem Alshagri, Shabeer Khan

**Affiliations:** 1 Department of Economics, Faculty of Political Sciences, Sakarya University, 54050 Sakarya, Turkey; 2 Department of Finance and Banking Affairs, Faculty of Economics, Ghazni University, Ghazni, Afghanistan; 3 Department of Economics, King Saud University, Riyadh, Saudi Arabia; 4 Department of Islamic Economics and Finance, Faculty of Political Science, Sakarya University, 54050 Serdivan, Turkey; 5 Economics Department, King Saud University, Riyadh, Saudi Arabia; 6 College of Business, Al Yamamah University, Riyadh, Saudi Arabia

**Keywords:** Natural resources, Environmental quality, Globalization, Renewable energy, OIC countries, Climate-change policy, Climate-change ecology, Environmental economics, Ecology, Environmental social sciences

## Abstract

Climate change has intensified environmental challenges globally, with greenhouse gas emissions rising more rapidly in Organization of Islamic Cooperation (OIC) countries than the global average. Over half of the OIC member states face high climate vulnerability due to limited institutional and technological capacity for mitigation and adaptation. This study makes a novel contribution by analyzing the drivers of environmental quality in 36 OIC countries from 1996 to 2020 using the load capacity factor (LCF), a holistic indicator that balances ecological demand and biocapacity supply. Unlike prior studies that focus narrowly on CO₂ emissions, LCF provides a broader measure of sustainability. The analysis is stratified by income levels (high, upper-middle, and lower-middle income) to uncover income-specific dynamics. The CS-ARDL approach was employed to estimate both short- and long-run relationships. The findings reveal that economic expansion improves environmental quality in high-income but degrades it in upper- and lower-middle-income groups. Renewable energy consumption consistently enhances environmental sustainability across all income groups, while natural resource dependence undermines it. Economic globalization improves environmental quality in high-income but has adverse effects in middle-income economies. Notably, the rule of law emerges as a strong and consistent driver of environmental improvement across all groups. These findings underscore the need for income-specific strategies that prioritize renewable energy investment, promote sustainable natural resource management, strengthen institutional quality, and integrate environmental objectives into globalization and trade policies.

**Supplementary Information:**

The online version contains supplementary material available at 10.1038/s41598-025-16872-y.

## Introduction

Environmental balance, prosperity, and security are central to the Sustainable Development Goals (SDGs). For example, SDG 7 emphasizes the need for accessible, reliable, and sustainable energy solutions. Sustainability hinges on the interplay between economic and environmental aspects, both playing crucial roles in fostering long-term development and resilience^1^. These two dimensions frequently exhibit an inverse relationship, as promoting economic growth without compromising environmental integrity remains difficult. This challenge is largely because sustained economic development in many nations heavily relies on fossil fuels, which, in turn, adversely affects efforts to enhance environmental quality^2^. Despite this, several developed nations have adopted sustainable energy alternatives, enabling them to mitigate the environmental damage caused by fossil fuel usage through advanced technology and substantial long-term R&D investments.

The need to identify drivers that simultaneously improve environmental quality and support economic performance is vital. Given the substantial impact of macroeconomic factors on environmental quality, the formulation of robust environmental policies is imperative. Earlier research has investigated a range of factors that may positively influence environmental conditions. For example, studies have explored the effects of technological innovations and renewable energy^3^, human capital^2,4^, nuclear energy and R&D^5,6^, and globalization^7,8^ on environmental changes across different nations and regions. However, there remains a clear gap in the literature regarding how renewable energy consumption (REC), natural resources (NR), and economic globalization (EGL) affect environmental quality in the Organization of Islamic Cooperation (OIC) countries, which comprise a unique group of resource-dependent, economically diverse, and environmentally vulnerable nations. Most existing studies have excluded these geopolitical blocs, despite its growing ecological footprint and developmental importance.

**Fig. 1:**
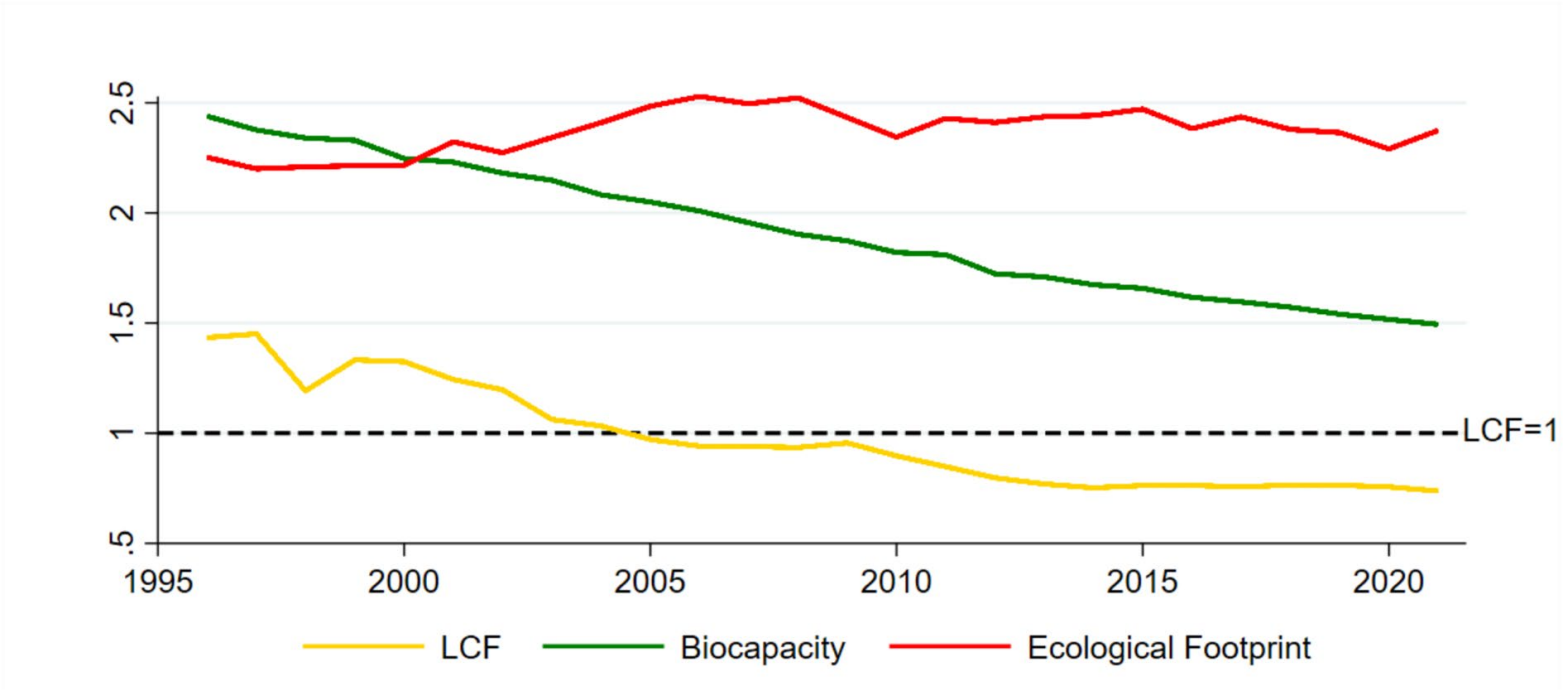
Trends in environmental quality: biocapacity, ecological footprint, and LCF (global hectares per capita) in OIC countries. *Sources:*^32^.

The current body of literature has measured environmental quality through several different factors. For example, CO_2_ emissions have been considered by researchers such as^9,10^, and^11^. However, CO_2_ emissions only capture air pollution, providing a limited measure of environmental quality. Consequently, some researchers, like^2,12,13^ have utilized the ecological footprint (EF) to gauge environmental degradation. EF captures air, soil, and water pollution, offering a broader measurement compared to CO_2_. EF is a measure of human consumption of biological resources, making it possible to compare between resource use and the availability of biologically productive marine and land areas. It represents the demand for ecosystem products and services such as natural capital^14^. Conversely, biocapacity evaluates an ecosystem’s regenerative ability to produce resources and manage waste, reflecting its capacity to supply what is needed to satisfy consumption demands.^15^. However, the EF metric fails to consider the supply capacity of ecosystems. This study addresses this limitation by employing the Load Capacity Factor (LCF), a holistic index that incorporates both environmental demand (EF) and supply (biocapacity), introduced by Siche et al.^16^ and adopted in recent works^15,17–19^. The LCF is computed as biocapacity divided by EF (Biocapacity *${EF}^{-1}$). An LCF value of 1 represents the sustainability threshold. When the LCF exceeds 1, it indicates sustainable environmental quality, suggesting that the current environmental resources are adequate to fulfill human demands^4^. Conversely, an LCF below 1 signifies unsustainable environmental quality. The LCF acts as a holistic environmental indicator, capturing both the demand side—showcasing human impacts on land, water, and air quality—and the supply side, which reflects nature’s capacity to manage these pressures. The environmental sustainability performance of the OIC countries from 1996 to 2021, as depicted in Fig. 1, shows a concerning trajectory. While the Ecological Footprint has risen sharply after the 2000s, biocapacity has steadily declined. This has led to a drop in the LCF below the sustainability threshold since 2005, indicating a continuous deterioration in environmental sustainability across OIC member states.

This research investigates the roles of REC, natural resources, and economic globalization in shaping environmental excellence, using the LCF as an indicator. NR significantly contributes to the economic development of many nations. However, the same resources that fuel economic growth can also harm the environment through their extraction and use in producing goods. The process of extracting and utilizing NR, including fossil fuels, land, water, soil, and minerals, results in substantial environmental degradation and the gradual exhaustion of these critical resources. Moreover, the extraction process itself requires substantial NR and is energy-intensive, resulting in high greenhouse gas (GHG) emissions^15^. Consequently, while NR facilitates economic activities and energy access, it also causes several ecological externalities. Existing literature shows varied impacts of NR on environmental changes. Some studies^20–22^ have concluded that NR negatively impact environmental quality, mainly because of outdated extraction and processing technologies. In contrast, other studies^13,23^ indicates that NR can positively impact environmental quality by decreasing reliance on fossil fuels and adopting innovative technologies.

Energy use has played an important role in driving economic expansion in various nations. Nevertheless, its benefits come at the cost of negative effects on environmental excellence. In response to these challenges, governments are showing growing interest in renewable energy as a strategy to alleviate environmental harm^3^. Solar, biofuel, hydroelectric power, and wind are sources of renewable energy that offer low-carbon solutions to satisfy energy demands while lowering the ecological footprint. In contrast to fossil fuels, these renewables generate electricity without emitting GHGs that cause air pollution^22^. Renewable energy utilization alleviates the overexploitation and combustion of fossil fuels like coal and petroleum^20^. Despite numerous research highlighting the environmental benefits of REC, some research^24,25^ have shown that REC has sometimes increased environmental deterioration. Economic globalization refers to the process of integrating countries internationally and fostering competition by lowering trade and capital flow barriers across borders. While the world confronts numerous climate change risks during globalization, the environmental effects of EGL are emerging as a significant issue^26^. Through globalization, the transfer of eco-friendly technologies from developed to developing countries can significantly enhance environmental outcomes^27^. In a globalized context, advance technology and expertise from more developed nations can stimulate the utilization of clean energy and improve overall environmental quality^28^. Conversely, globalization can harm environmental quality by facilitating the movement of pollution-heavy industries to developing nations from developed ones, where production costs are lower.^10^. Furthermore, the inflow of foreign investment induced by globalization may incentivize the overexploitation of natural resources to maximize profits, further compromising environmental sustainability^29^. Furthermore, the expanded availability of energy-intensive technologies driven by globalization may exacerbate pollution and environmental deterioration^30^.

**Fig. 2:**
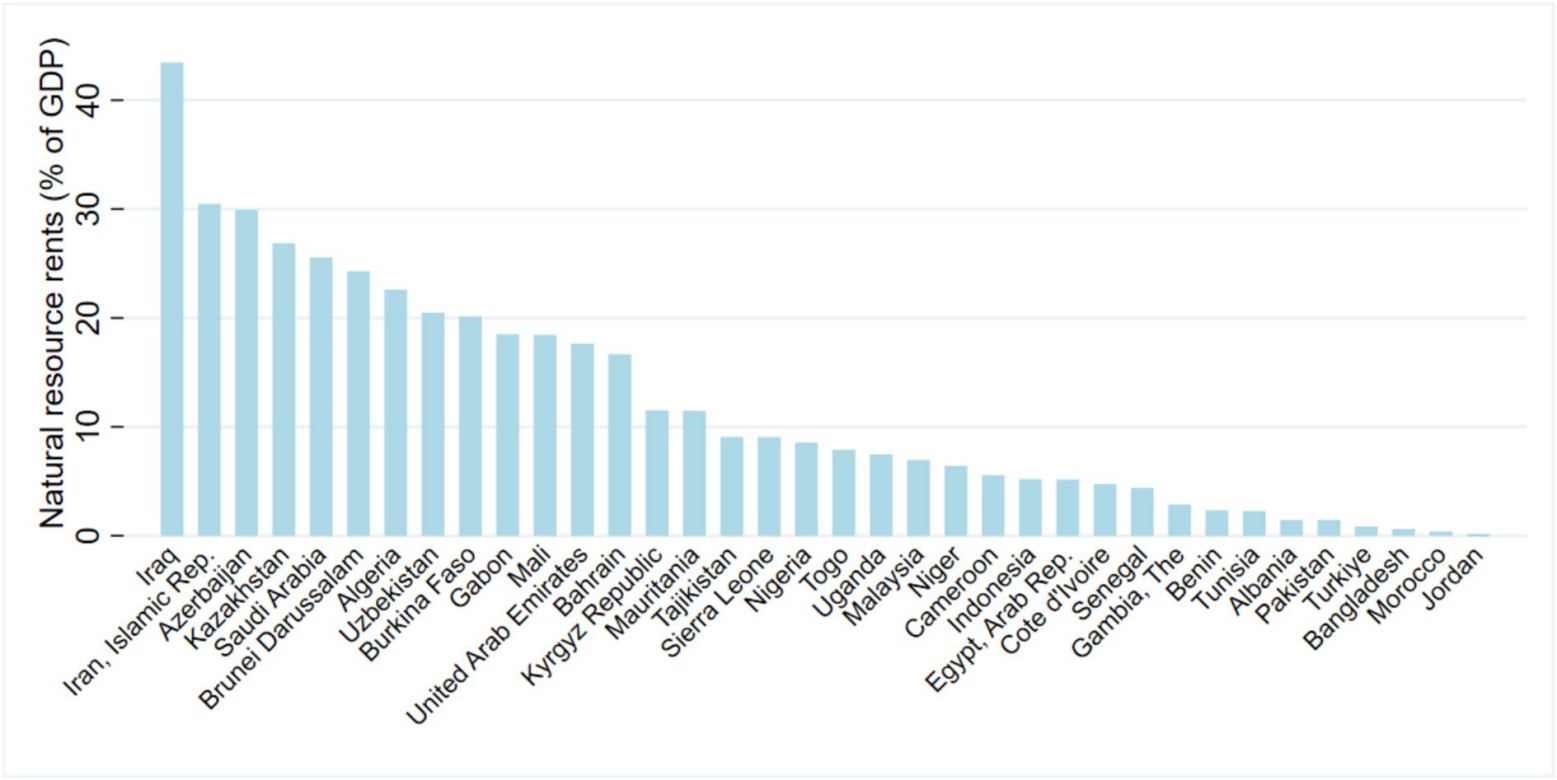
Proportion of natural resource rents to GDP in OIC nations, 2021. *Source*:^33^.

The decision to focus on OIC countries in this study stems from their significant vulnerability to environmental degradation, largely due to their reliance on fossil fuels and insufficient economic diversification. The OIC countries, comprising 52 Muslim-majority nations across four continents, are heavily rely on natural resources, especially non-renewable subsoil assets, which significantly contribute to their GDP. According to the OIC Environment Report 2023, the value of these resources within OIC countries experienced a near-doubling (90%) between 1998 and 2018, culminating in a total value of US$21.9 trillion. The primary driver of this growth was a substantial 137.4% increase in non-renewable, subsoil assets. In 2018, natural resource rents constituted an average of 13.8% of OIC countries’ GDP, with oil rents being a major component. The average share of natural resource rents in GDP for individual OIC member states depicted in Fig. 2. Furthermore, OIC countries face substantial environmental pressures, as reflected in their low average Environmental Performance Index (EPI) score of 35.7 in 2022, compared to 40.7 for developing countries and 60.6 for developed countries. Deforestation rates have increased from 0.27% during 2000–2010 to 0.44% during 2010–2020. Air pollution poses a critical health risk, with 1.6 million deaths attributed to air pollution in 2022, significantly exceeding the global average, with a death rate of 131 per 100,000 people compared to the global average of 86. Moreover, while global GHG emissions rose by 53% from 1990 to 2019, OIC countries experienced a 91% increase, largely due to economic growth and population expansion^31^. The exploitation and consumption of NR in these nations have led to environmental harm, including pollution and habitat loss. Tackling this challenge is essential to advancing SDG 7, which focuses on affordable and clean energy. Against this backdrop, this study aims to explore the following research question: How do renewable energy use, natural resource rents, and economic globalization shape environmental quality across different income-level OIC countries, in the short and long term? To address this question, the study formulates and tests the following hypotheses:

### H1

Renewable energy use enhances environmental sustainability across all income-level OIC countries.

### H2

Higher dependence on natural resource rents deteriorates environmental quality in OIC countries, regardless of income level.

### H3

Economic globalization enhances environmental quality across all income-level OIC countries.

Building on this research question and these hypotheses, the study examines the environmental quality in OIC countries between 1996 and 2021, using the LCF as an indicator. The key explanatory variables are renewable energy, natural resources, and economic globalization, while economic performance, role of law, and population serve as control variables, given their potential influence as main contributors to environmental degradation. As the primary model, the study utilizes the Cross-Sectionally Augmented Autoregressive Distributed Lag (CS-ARDL) approach, with FMOLS, DOLS, and ECM models employed for the purposes of robustness checks. The CS-ARDL method provides advantages over other conventional models by allowing both short- and long-term estimations while addressing slope heterogeneity, cross-sectional dependency (CSD), and mixed integration orders of variables.

This research seeks to make several important contributions to the literature. First, it examines the influence of REC, NR, EGL on the environmental quality of OIC nations, making it the first to focus specifically on this diverse and underexplored group. Second, it employs the LCF as a comprehensive indicator of environmental sustainability, which captures both the supply and demand dimensions of ecological systems, thereby addressing limitations in studies that rely solely on traditional indicators like CO₂ emissions or ecological footprint. Third, the study applies the CS-ARDL model, an advanced econometric technique that accounts for cross-sectional dependence (CSD), heterogeneity, and omitted variables bias in panel data. This methodological approach yields robust short- and long-run estimates, enhancing the reliability of estimation. Fourth, to capture heterogeneity and derive more realistic and policy-relevant insights, the analysis is conducted across three income-based subgroups within OIC countries: high-income, upper-middle-income, and lower-middle-income groups. Finally, in addition to these empirical and methodological advancements, the study provides tailored policy recommendations for OIC policymakers, offering actionable insights to help these nations achieve the SDGs, promote inclusive economic growth, and transition toward environmentally sustainable development paths.

The structure of the research is as follows: Sect. “Literature review” reviews the literature, Sect. “Method and data” details the methodology and data used, Sect. “Results and discussion” presents the findings along with the discussion, and Sect. “Conclusion and policy recommendations” concludes by offering policy recommendations.

## Literature review

This part of the paper examines prior studies investigating the link between renewable energy consumption (REC), natural resources (NR), and economic globalization (EGL) with environmental quality. The analysis is structured into three distinct contexts for better comprehension.

### NR and environmental quality

The environmental implications of natural resource rents have been widely debated, with studies reporting mixed outcomes. While many emphasize the detrimental effects of NR dependence, others reveal more nuanced or even beneficial outcomes. Numerous studies indicate that high dependence on NR deteriorates environmental quality. For instance, Sun et al.^34^ found that natural resources increase ecological footprint (EF) in G-11 countries, while^20^ confirmed similar findings in BRICS nations. Erdoğan et al.^27^ identified a positive relationship between natural resource abundance and EF in Sub-Saharan Africa, whereas Zhang et al.^22^ showed that natural resource revenues degrade ecological quality in resource-rich countries. Dao et al.^35^, analyzing 27 countries, concluded that excessive reliance on natural resource rents generally leads to environmental deterioration. However, some studies suggest that natural resources may improve environmental quality. For example, Sun et al.^23^ examined 17 APEC economies and found that natural resources enhance environmental quality by improving LCF. Similarly, Khan et al.^13^ demonstrated that natural resources reduce both EF and CO₂ emissions in the U.S. Notably, some studies argue that the environmental effects of natural resources are asymmetric or context-dependent. For instance, Ullah and Lin^36^ observed in Pakistan that natural resources increase EF while reducing CO₂ emissions, highlighting the role of policy and technological factors in shaping these outcomes.

### Environmental impacts of renewable energy consumption

Renewable energy consumption is widely regarded as a pivotal strategy for achieving environmental sustainability. The literature consistently shows that REC enhances environmental quality by reducing GHG emissions and supporting green innovation. Unlike fossil fuels, renewable sources emit minimal or no carbon, thereby improving air quality and mitigating climate change. Several empirical studies confirm the beneficial environmental impacts of REC. Mohamed et al.^37^ found that REC reduces CO₂ emissions in Malaysia in the long run, while Khan et al.^38^ showed that REC has a strong negative effect on CO₂ emissions, particularly in high-emission SAARC countries. REC alleviates environmental pressure by improving natural resource efficiency. Villanthenkodath and Pal^39^ demonstrated in India that REC reduces both EF and carbon intensity, while Jahanger et al.^3^ found that REC significantly contributes to natural resource conservation in the top 10 countries leading in sustainable development goals. From a green economic growth perspective, REC plays a pivotal role. Deshuai et al.^40^ revealed that REC positively affects green economic growth in China in both the short and long term. Similarly, Kazemzadeh et al.^41^, analyzing 64 countries, found that transitioning from fossil fuels to renewable energy reduces EF, fostering sustainable development.

While early studies primarily measured REC’s effects through carbon emissions, recent research has shifted focus to the LCF. Numerous regional studies confirm the positive impact of renewable energy investments on LCF. Teng et al.^42^ in major nuclear energy-producing countries, Aquilas et al.^43^ across 46 African nations, Ali et al. ^44^ in MINT economies, Wang et al.^45^ in selected Asian countries, Aydin and Erdem^46^ in 18 EU member states, and Caglar and Yavuz^47^ in 22 EU countries. However, not all findings are favorable. Altıntaş et al.^25^ reported that REC decreases LCF in Malaysia in both the short and long term, while Alola et al.^24^ found that REC increases EF in 16 EU countries, underscoring the context-dependent nature of these effects.

### Globalization and environment

The relationship between globalization and the environment is examined through three major theoretical frameworks, each yielding distinct empirical findings. The Triple Impact Model explains globalization’s environmental effects through scale, composition, and technique effects^48^. Studies emphasizing scale effects argue that globalization exacerbates environmental degradation by expanding production. Wang et al.^49^ found that globalization increases greenhouse gas emissions in G20 countries, while Awad and Saadaoui Mallek^50^ showed that economic globalization worsens environmental quality in SSA. Conversely, technique effects can mitigate environmental harm through technology diffusion. Erdoğan et al.^27^ demonstrated that FDI spreads eco-friendly technologies in SSA, reducing EF. Padhan et al.^51^ confirmed that FDI lowers carbon emissions and promotes green technology in OECD countries. However, institutional quality matters, Gaies et al.^26^ found that weak environmental regulations in MENA countries allow globalization to increase CO₂ emissions.

The environmental effects of globalization, particularly through FDI, are also examined within two contrasting hypotheses. Pollution Haven Hypothesis (PHH) argues that multinational corporations relocate to countries with lax environmental standards^52^. Supporting PHH, Bekun et al.^53^ showed that FDI in Turkey leads to higher CO₂ emissions, and Kholil et al.^54^ found that FDI increased ecological footprint in ASEAN nations. Conversely, the Pollution Halo Hypothesis (PHEH) posits that FDI brings environmentally friendly technologies^55,56^. Supporting PHEH, Mert and Caglar^57^ found that FDI reduced CO₂ emissions in Turkey. Another major line of research on the globalization-environment nexus employs the EKC hypothesis. Originally proposed by Grossman and Krueger^58^ , the EKC hypothesis suggests an inverted U-shaped relationship between economic performance and environmental degradation. Shahbaz^59^ identified U- or inverted U-shaped relationships in Next-11 countries including Bangladesh, Iran, Philippines, Vietnam, and South Korea. Latif et al.^7^ confirmed the EKC hypothesis by identifying inverted U-shaped relationships between economic globalization and LCF in Asian countries. Leal et al.^10^ found that EKC holds OECD countries with high globalization levels. Jahanger^60^ highlighted positive effects of political globalization while noting adverse impacts of economic and social globalization. Farooq et al.^61^, using data from 180 countries, supported the EKC hypothesis but noted that economic globalization generally impairs environmental sustainability.

Recently, the Load Capacity Curve (LCC) was proposed to address EKC limitations, suggesting a U-shaped link between income and LCF^4^. Aydin et al.^8^ tested LCC in 10 high-tech EU countries, confirming it only for Spain, while globalization reduced LCF in Austria—highlighting the complexity of these relationships.

### Research gap

Despite the growing literature on the environmental effects of REC, NR, and globalization, significant gaps remain. Few studies have jointly examined these variables in the context of OIC countries, and even fewer have employed comprehensive environmental indicators like LCF, which captures both ecological demand and biocapacity. Methodologically, most existing research relies on traditional panel techniques, often overlooking advanced approaches such as the CS-ARDL, which account for cross-sectional dependence and heterogeneity. Moreover, the heterogeneity across income groups within the OIC has been largely ignored. To address these gaps, this study makes several contributions: it focuses specifically on OIC countries, uses a more holistic measure of environmental quality (LCF), applies robust econometric methods (CS-ARDL), and uniquely examines the environmental impacts across three income-based subgroups, offering more context-specific and realistic insights.

## Method and data

### Data and sources

**Fig. 3:**
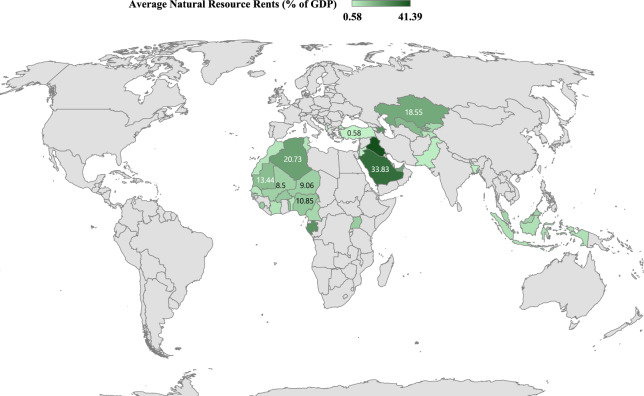
Geographical location of the OIC countries on the world map, according to their resource endowment.

This research investigates the influence of renewable energy consumption, natural resources, economic globalization, economic performance (GDP), role of law (RL), and population (POP) on the environmental performance of 36 selected member countries of the OIC from 1996 to 2021. To capture heterogeneity, the analysis stratifies these countries into high-, upper-middle-, and lower-middle-income groups. This income-based classification reveals notable differences in the environmental effects of the studied variables. The list of selected OIC countries, categorized by income level, geographic region, and resource endowment, is provided in Table 1. The selection of these countries was based on the availability of consistent and balanced panel data for the variables of interest over the study period. Only countries with complete and reliable data for all seven variables (LCF, NR, REC, EGL, GDP, RL, and POP) throughout the full 1996–2021 period were included. This criterion ensures robustness in estimation and comparability across cross-sections. Figure 3 presents the geographical distribution of these countries. Consistent with the recent literature, this study employs the LCF as the dependent variable to represent environmental quality. The LCF captures the supply and demand dimensions of the natural ecosystem, offering a thorough analysis of environmental quality. This approach contrasts with previous studies that consider just the demand aspect trough CO2 emissions or EF, by using the LCF, derived from dividing biocapacity by the EF. The study identifies NR, REC, and EGL as the main explanatory variables, with GDP, RL, and POP are considered as control variables due to their potential roles as the primary drivers of environmental degradation. The LCF data are collected from GFN^32^ , whereas EGL data are obtained from KOF^62^. Data for GDP and POP are collected from WDI^33^ and RL data are taken from WGI^63^. To tackle heteroscedasticity and non-linearity issues, and to facilitate the interpretation of coefficients as elasticities, the study applies natural logarithms to all variables. Table 2 provides variable descriptions along with their sources.

**Table 1:** OIC member countries classified by income level, region, and resource endowment.

Income	Region	Resource-rich	Resource-poor
High	MENA	Bahrain, Saudi Arabia, UAE	–
	Southeast Asia	Brunei Darussalam	–
Upper-middle	Central Asia	Azerbaijan, Kazakhstan, Uzbekistan	–
	Southeast Asia	Indonesia, Malaysia	–
	MENA	Algeria, Egypt, Iran, Iraq	Jordan, Morocco, Tunisia
	Europe/Asia	–	Türkiye, Albania
Lower-middle	Central Asia	Kyrgyz Rep., Tajikistan	–
	South Asia	–	Bangladesh, Pakistan
	SSA	Burkina Faso, Cameroon, Gabon, Mali, Mauritania, Niger, Nigeria, Sierra Leone, Togo, Uganda	Benin, Côte d’Ivoire, Gambia, Senegal

Income levels are based on the World Bank’s 2024 classification, where high-income countries have a GNI per capita above 13,935 USD, upper-middle-income countries fall between 4496 and 13,935 USD, and lower-middle-income countries range from 1136 to 4495 USD. Countries are considered resource-rich if their total natural resource rents (from oil, gas, or minerals) exceed 5% of GDP.

**Table 2:** Description and sources of variables.

Variable	Symbols	Unit	Sources
Load capacity factor	LCF	Biocapacity/ecological footprint (global hectares per capita)	^32^
Natural resources	NR	Share of total natural resource rents as a percentage of GDP	^33^
Renewable energy	REC	Proportion of renewable energy consumption in the total final energy consumption (%)	^33^
Economic growth	GDP	Constant US$ 2015, per capita	^33^
Economic globalization	EGL	KOF economic globalization index, scales 0–100 score	^64^
Population	POP	Total population, million people	^33^
Role of law	RL	Score from − 2.5 to + 2.5	^63^

### Model specification

Building on recent work of Işık et al.^3^, Ni et al.^15^ and Pata et al.^21^, which examine environmental improvement using the LCF, this research investigates the effects of NR, REC, EGL, POP, RL, and GDP, on LCF in OIC member states. Grounded in theoretical and empirical studies, the baseline model for this research is defined as below:

1 $$LFC = f\left( {{\text{NR}},{\text{REC}},{\text{EGL}},{\text{GDP}},{\text{RL}},{\text{POP}}} \right)$$

here LCF serves as the dependent variable, while NR (natural resources), REC (renewable energy consumption), and EGL (economic globalization) as the independent variables. POP (population), RL (role of law) and per capita GDP are included as control variables. To empirically test the model, Eq. (1) is restructured into the following regression model:

2 $$lnLCF_{it} = \alpha_{it} + \beta_{1,it} lnNR_{it} + \beta_{2,it} lnREC_{it} + \beta_{3,it} lnEGL_{it} + \beta_{4,it} lnGDP_{it} + \beta_{5,it} lnRL_{it} + \beta_{6,it} lnPOP_{it} + \varepsilon_{it}$$

here $\beta$’s represents the coefficients for the regressors corresponding to the $i$ cross-section over the time $t$, $\alpha$ is the intercept, and $\varepsilon$ is the error term.

### Econometric procedure

**Fig. 4:**
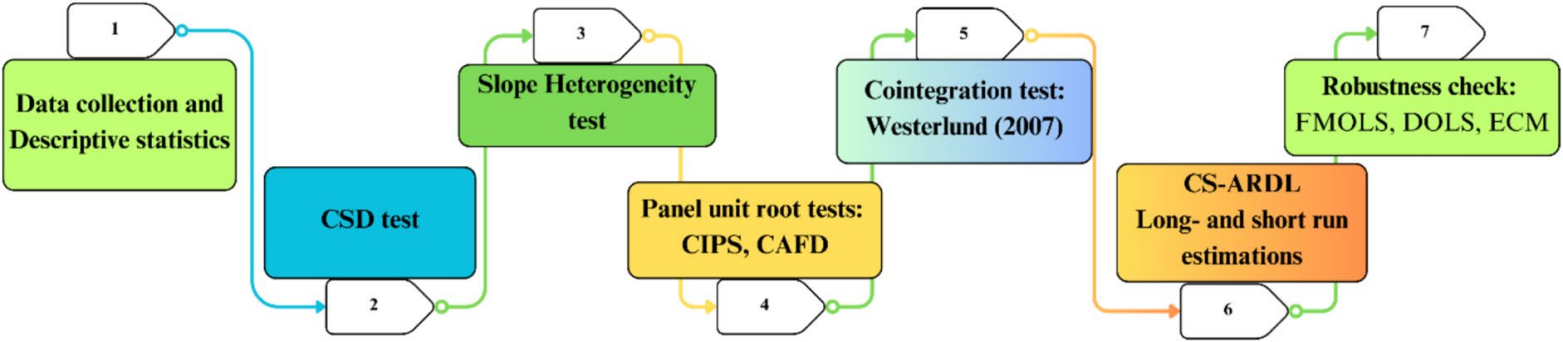
Flow of econometric steps.

The study used several estimation techniques to assess the environmental quality of OIC member nations. Figure 4 provides a summary of these seven empirical techniques steps, which are designed to analyze the effects of NR, REC, EGL, POP, RL, and GDP on environmental quality.

#### Cross-sectional dependence (CSD) test

In recent decades, increased economic, cultural, and social interactions among countries have led to a situation where shocks in one country can potentially affect others. This raises concerns about CSD in our dataset. To address this challenge, our first task is to evaluate the presence of CSD to identify the most suitable unit root tests. For this purpose, we employed CSD test introduced by Pesaran^65^ for OIC member countries. A statistical significance result allows us to reject the H_0_ hypothesis, indicating that there are CSD issues in the dataset. The CSD test, which designed to handle large N (cross-sections) is computed as follows:

3 $$CD = \sqrt {\frac{2T }{{N\left( {N - 1} \right)}}} \left( {\mathop \sum \limits_{i = 1}^{N - 1} \mathop \sum \limits_{j = i + 1}^{N} \hat{\rho }_{ij} } \right) \sim N\left( {0, 1} \right) i, j$$

#### Slope heterogeneity (SH) test

In panel analysis, differences in development levels, resource richness, and environmental sustainability characteristics among countries can lead to variations in coefficient slopes. This variability necessitates a slope heterogeneity test prior to regression analysis to avoid potentially misleading results. Accordingly, we performed the slope heterogeneity test introduced by Pesaran and Yamagata^66^, as outlined below;

4 $$\tilde{\Delta }_{SH} = (N)^{\frac{1}{2}} (2k)^{{ - \frac{1}{2}}} \left( {\frac{1}{N}\tilde{S} - k} \right)$$

5 $$\tilde{\Delta }_{ASH} = (N)^{\frac{1}{2}} \left( {2k\left( {\frac{T - k - 1}{{T + 1}}} \right)} \right)^{{ - \frac{1}{2}}} \left( {\frac{1}{N}\tilde{S} - k} \right)$$

where $\tilde{\Delta }_{SH}$ represents the delta tilde, while $\tilde{\Delta }_{ASH}$ denotes the adjusted delta tilde. The H_0_ test is that the slopes are homogeneous.

#### Panel unit root test

Upon identifying CSD in the data, we employed 2nd generation unit root tests to determine the existence of a unit root in the panel data by testing the null hypothesis of non-stationarity. Traditional panel stationarity tests are insufficient for dealing with CSD in panel datasets. To overcome these limitations, we conducted the CIPS and CADF tests based on the methodology proposed by Pesaran^67^. The econometric equations corresponding to the regression of CADF are outlined as follows:

6 $$\Delta y_{it} = \alpha_{i} + \beta_{i} y_{i,t - 1} + c_{i} \overline{y}_{t - 1} + \mathop \sum \limits_{j = 0}^{p} d_{ij} \Delta \overline{y}_{t - j} + \mathop \sum \limits_{j = 1}^{p} \theta_{ij} \Delta y_{i,t - j} + e_{it}$$

Furthermore, the CIPS is determined by averaging the CADF values for each individual $i$ as follows:

7 $$CIPS = N^{ - 1} \mathop \sum \limits_{i = 1}^{N} t_{i} \left( {N,T} \right)$$

where, the term $t_{i} \left( {N,T} \right)$, represents the CADF statistic in the following equation:

8 $$CIPS = N^{ - 1} \mathop \sum \limits_{i = 1}^{N} CADF_{i}$$

#### Westerlund cointegration test

Given the CSD and slope heterogeneity in the data, it is essential to apply a heterogeneous estimating method to determine long-run cointegration. Therefore, the Westerlund^68^ cointegration test is conducted, as it handles CSD and heterogeneity and remains robust against serial correlation in the data^2^. This test is grounded in the null hypothesis (H0), which assumes no cointegration. The following four formulas are used to estimate cointegration in the group statistics (Ga and Gt) and the panel statistics (Pa and Pt):

9 $$G_{t} = \frac{1}{N}\mathop \sum \limits_{i - 1}^{N} \frac{{\varphi_{i} }}{{SE\left( {\hat{\varphi }_{i} } \right)}}$$

10 $$G_{a} = \frac{1}{N}\mathop \sum \limits_{i - 1}^{N} \frac{{T\varphi_{i} }}{{\mathop \varphi \limits^{\prime }_{i} \left( 1 \right)}}$$

11 $$P_{t} = \frac{{a\varphi_{i} }}{{SE\left( {\hat{\varphi }_{i} } \right)}}$$

12 $$P_{a} = T\hat{\varphi }_{i}$$

#### CS-ARDL approach

In the subsequent stage, we employed the Cross-Sectionally Augmented ARDL (CS-ARDL) method introduced by Chudik and Pesaran^69^ to analyze short- and long-run estimations. CS-ARDL builds upon the ARDL model by including cross-sectional means of the variables, allowing it to account for CSD. This method is particularly well suited to the characteristics of our dataset for several reasons. First, it addresses CSD, which was confirmed by preliminary tests among the panel units. The CS-ARDL model incorporates cross-sectional averages of both the dependent and explanatory variables to effectively control for this issue. Second, it accommodates mixed orders of integration, as our variables exhibit a combination of I(0) and I(1) stationarity levels. Unlike conventional panel estimation techniques, CS-ARDL remains robust in the presence of such mixed integration orders and non-stationarity. Third, the model allows for heterogeneous slope coefficients across countries, an essential feature considering the economic and environmental diversity among the OIC member states. Fourth, it mitigates endogeneity and omitted variable bias by incorporating lagged dependent and independent variables as well as cross-sectional means. This structure helps account for potential reverse causality and unobserved common factors, thereby enhancing the reliability of the estimations^47^. The CS-ARDL equation is presented as follows;

13 $$Y_{it} = \alpha_{i} + \mathop \sum \limits_{l = 1}^{{p_{y} }} \varphi_{i1} Y_{i,t - 1} + \mathop \sum \limits_{l = 0}^{{p_{x} }} \beta_{i1}{\prime} X_{i,t - 1} + \mathop \sum \limits_{l = 0}^{{p_{{\overline{z}}} }} \gamma_{i1}{\prime} \overline{Z}_{t - 1} + \varepsilon_{it}$$

where $\alpha_{i}$ is a constant term, $Y_{it}$ denotes the dependent variable, referred to as LCF. $X_{it} = \left( {NR_{it} ,REC_{it} ,EGL_{it} ,GDP_{it} ,POP_{it} } \right)$ indicates all independent variables, ${p}_{y}$, ${p}_{x}$ and ${p}_{\overline{z} }$ are the lags numbers. $\overline{Z }$ is the mean value of the independent and dependent variables $({\overline{Z} }_{t-1}= {\overline{Y} }_{i,t-1}, {\overline{X} }_{i,t-1})$. $i$ stands for cross-section (i.e., country), where $i=\text{1,2},\text{3,4},\dots ,36$, for time $t=1,$. $\varepsilon_{it}$ is the error term. The long-run individual average coefficients derived from the short-run coefficients ($\hat{\varphi }_{i1} ,{ }\hat{\beta }_{i1} )$ based on Eq. (13) is presented in Eq. (14)

14 $$\hat{\theta }_{CS - ARDL,i} = \frac{{\mathop \sum \nolimits_{l = 0}^{{p_{x} }} \hat{\beta }_{i1} }}{{1 - \mathop \sum \nolimits_{l = 1}^{{p_{y} }} \hat{\varphi }_{i1} }},$$

The mean long-run effects are based on the mean group estimation as follows;

15 $$\widehat{{\overline{\emptyset }}}_{MG} = \frac{1}{N}\mathop \sum \limits_{i = 0}^{N} \hat{\theta }_{CS - ARDL,i}$$

The short-run coefficient estimations are as bellows;

16 $$\Delta Y_{it} = \gamma_{i } \left( { Y_{it - 1} - \theta_{i} X_{it - 1} } \right) - \mathop \sum \limits_{l = 1}^{{P_{y} - 1}} \varphi_{i1} \Delta Y_{i,t - 1} + \mathop \sum \limits_{l = 0}^{{P_{x} }} \beta_{i1}{\prime} \Delta X_{i,t - 1} + \mathop \sum \limits_{l = 0}^{{P_{{\overline{z}}} }} \gamma_{i1}{\prime} \overline{Z}_{t - 1} + \varepsilon_{it}$$

where $$\Delta_{i} = t - \left( {t - 1} \right).$$

The CS-ARDL estimation’s validity is confirmed by a negative and significant Error-Correction Term (ECT), indicating that the model is stable and converging towards a long-term equilibrium^70^.

To ensure robustness checks, Dynamic OLS (DOLS) and Fully Modified OLS (FMOLS) methods were applied to assess the long-run associations, while the Error Correction Model (ECM) was used to capture short-term dynamics among the variables. Furthermore, we utilized the Dumitrescu and Hurlin^71^ Granger causality test to explore the direction of the relationship between each variable.

## Results and discussion

### Descriptive statistics, correlation matrix, and multicollinearity test

This section starts with an exploration of the dataset’s descriptive statistics and correlation matrix. Descriptive statistics for 36 OIC nations, based on 936 observations for each variable, are presented in Table 3. The mean LCF is − 0.59, with values ranging from − 2.84 to 3.14, and a moderate right skew (0.40). GDP averages 7.83, NR 1.87, REC 2.39, EGL 3.84, and POP 16.52, each with varying degrees of spread. NR and REC are left-skewed, while EGL and POP are nearly symmetric. Notably, RL shows a mean of 0.16 but with extreme left skew (− 3.38) and high kurtosis (30.92), indicating large disparities in governance across countries.

**Table 3:** Descriptive statistics.

	N	Mean	SD	Min	Max	Skewness	Jarque–Bera	Kurtosis
lnLCF	936	− 0.59	0.94	− 2.84	3.14	0.4	3.145	4.96
lnGDP	936	7.83	1.22	5.91	11.04	0.71	11.04	2.79
lnNR	936	1.87	1.31	− 3.15	4.18	− 0.84	4.179	3.51
lnREC	936	2.39	2.22	− 2.3	4.57	− 0.9	4.569	2.46
lnEGL	936	3.84	0.30	2.77	4.47	− 0.14	4.474	2.88
lnPOP	936	16.52	1.47	12.61	19.44	− 0.21	19.44	2.92
lnRL	936	0.16	0.60	− 7.12	1.01	− 3.38	1.014	30.92

To evaluate multicollinearity among the independent variables, both the correlation matrix and the Variance Inflation Factor (VIF) were analyzed in this study. Table 4 presents the correlation matrix for the full sample. The LCF is negatively correlated with GDP (− 0.43), EGL (− 0.40), RL (− 0.13), and POP (− 0.14), suggesting that higher income, globalization, and institutional quality are generally associated with lower environmental degradation. Conversely, LCF shows a strong positive correlation with REC (0.66), indicating that greater renewable energy use is linked to improved environmental performance. Correlations among other variables are generally modest, with no immediate multicollinearity concerns. Furthermore, the VIF values range from 1.17 to 4.19, with all values below the commonly used threshold of 5. This indicates that no single independent variable has an inflated variance due to its linear relationship with other variables. Given that the VIF values are under 5, it indicates that multicollinearity does not pose issues in the model^18^. Additionally, the 1/VIF values, ranging from 0.23 to 0.85, further support the absence of multicollinearity, as values closer to 1 indicate less concern about collinearity. The descriptive statistics and correlation matrices for the three income-level groups are presented in Supplementary Tables S1 and S2 online, respectively.

**Table 4:** Correlation matrix and VIF test results.

Variables	lnLCF	lnGDP	lnNR	lnREC	lnGL	lnPOP	lnRL	VIF	1/VIF
lnLCF	1.00							4.19	0.238408
lnGDP	− 0.43*	1.00						1.36	0.734095
lnNR	− 0.02*	0.32*	1.00					3.36	0.297350
lnREC	0.66*	− 0.82*	− 0.42*	1.00				1.93	0.516809
lnEGL	− 0.40*	0.65*	0.09*	− 0.51*	1.00			1.17	0.854487
lnPOP	− 0.14*	− 0.21*	− 0.16*	0.18*	− 0.32*	1.00		1.35	0.742671
lnRL	− 0.13*	0.36*	− 0.14*	− 0.18*	0.37*	− 0.22*	1.00		
Mean VIF								2.23	

*denotes 1% significance level.

### Results of CSD and slope heterogeneity tests

Pesaran’s^65^ CSD test reveals significant interdependence among all variables for the OIC nations. Table 5 presents these findings, where the CD-test decisively rejects the independence hypothesis at the 1% significance level, highlighting substantial inter-country correlations. The results shown in Table 5, reveal substantial slope heterogeneity across the OIC member countries. The examination of Delta and adjusted Delta values indicates significant heterogeneity in the slopes of independent variables across nations, rejecting the homogeneity hypothesis at a 1% significance level. The outcome from the CSD and slope heterogeneity tests underscore the limitations of traditional testing methods for our data. Consequently, this research employed more advanced second-generation techniques for unit root and cointegration testing in the subsequent analysis.

**Table 5:** CSD tests results.

	Full smple		High-income		Upper middle		Lower middle	
Variable	CD-test	*P* value	CD-test	*P* value	CD-test	*P* value	CD-test	*P* value
lnLCF	69.710	0.000	8.840	0.000	14.480	0.000	48.830	0.000
lnGDP	60.110	0.000	1.340	0.180	45.950	0.000	32.770	0.000
lnNR	34.540	0.000	10.810	0.000	23.410	0.000	10.060	0.000
lnREC	26.740	0.000	3.610	0.000	3.550	0.000	28.260	0.000
lnEGL	27.350	0.000	3.440	0.001	13.530	0.000	10.320	0.000
lnPOP	112.460	0.000	12.400	0.000	37.790	0.000	59.240	0.000
lnRL	2.020	0.043	2.720	0.006	1.47	0.142	− 0.98	0.327

Slope heterogeneity test	Delta		Delta		Delta		Delta	
Statistics								
$\widetilde{\Delta }$	18.238	0.000	3.637	0.000	10.270	0.000	10.677	0.000
$\widetilde{\Delta }$Adjusted	21.919	0.000	4.371	0.000	12.343	0.000	12.832	0.000

### Results of stationarity and cointegration tests

To account for the presence of CSD in our model, we conducted Pesaran’s^67^ 2nd-generation panel unit root tests, particularly the CIPS and CADF tests. The results, shown in Table 6, reveal the order of integration for each variable analyzed in the study. The LCF, EGL, and RL are stationary in their level forms, with significant test statistics for both tests. In contrast, GDP, NR, POP, and REC exhibit non-stationarity at their levels but attain stationarity upon first differencing, classifying them as integrated of order one, I(1). This highlights a mixed integration order among the variables. The findings of the Westerlund^68^ bootstrap cointegration test, presented in Table 7, provide robust evidence of cointegration. Both the Gt and Pt statistics are significantly negative, with the *p* values and Z-values rejecting the null hypothesis of no cointegration. In particular, the robust *p* values further strengthen these findings. Collectively, these results imply a long-run equilibrium link among the variables analyzed. Given the confirmed long-run co-integration, along with the existence of CSD and varying slopes identified in earlier tests, the panel CS-ARDL method is deemed an appropriate econometric method for further analysis.

**Table 6:** Panel unit root tests results.

Variable	CIPS				CADF				Outcome
	Level	1st difference			Level		1st difference		Order of integration
	Constant	Constant and trend	Constant	Constant and trend	Constant	Constant and trend	Constant	Constant and trend	
lnLCF	− 2.714***	− 3.067***	−	−	− 2.613***	− 2.724***	−	−	I (0)
lnGDP	− 1.591	− 1.694	− 3.882***	− 4.291***	− 2.063**	− 2.237	− 2.869***	− 3.328***	I (1)
lnNR	− 1.914	− 2.502	− 4.242***	− 4.271***	− 2.233***	− 3.189***	−	−	I (1)
lnREC	− 0.875	− 1.512	− 4.025***	− 4.268***	− 1.063	− 1.567	− 2.903***	− 3.224***	I (1)
lnEGL	− 2.261***	− 2.574*	− 4.803***	− 4.848***	− 2.146***	− 2.514*	−	−	I (0)
lnPOP	− 1.712	− 1.121	3.65***	4.013***	− 2.561***	− 2.470	− 2.306***	− 2.763***	I (1)
lnRL	− 2.579***	− 3.625***	− 5.845***	− 5.993***	− 2.279***	− 3.345***	−	−	I (0)

***, **, and *denote the significance level at 1%, 5%, and 10%, respectively.

**Table 7:** Results of Westerlund bootstrap cointegration test.

Stat	Value	Z value	*P* value	Robust *P* value
Gt	− 2.387	− 3.968	0.000	0.000
Ga	− 0.610	0.632	0.271	0.000
Pt	− 11.703	− 2.900	0.002	0.030
Pa	− 5.395	− 1.069	0.143	0.060

### Results of CS-ARDL methods

In this research we utilized the CS-ARDL estimation approach to analyze the short- and long-run influence of renewable energy, natural resources, economic globalization, economic performance, role of law, and population on the LCF as a measure of environmental improvement in OIC nations from 1996 to 2021. The CS-ARDL model is particularly well-suited for this panel data analysis as it effectively addresses key empirical challenges, including cross-sectional dependence, endogeneity, reverse causality, and heterogeneity across countries. This methodological strength enhances the robustness and reliability of the estimated relationships. To account for structural heterogeneity in economic and environmental dynamics, the estimation results are disaggregated into the full sample and three income-based subgroups: high-, upper-middle-, and lower-middle-income countries. The model distinguishes between long-run and short-run effects, providing a comprehensive understanding of how various factors influence environmental quality, proxied by LCF. The findings presented in Table 8 illustrate important differences across income groups. The ECT coefficients are statistically significant and negative across all groups, with values of − 0.3285 for the full sample, − 0.3494 for high-income, − 0.4764 for upper-middle-income, and − 0.2870 for lower-middle-income countries. These values confirm the existence of a stable long-run equilibrium relationship between the explanatory variables and LCF in all cases. Moreover, the magnitude of the ECT coefficients indicates the speed of adjustment toward equilibrium following short-run deviations. Specifically, approximately 32.85% of disequilibrium is corrected each period in the full sample, 34.94% in the high-income group, 47.64% in the upper-middle-income group, and 28.70% in the lower-middle-income group. The faster adjustment observed in upper-middle-income countries suggests a more responsive environmental adjustment mechanism, potentially reflecting more active policy interventions or greater sensitivity to environmental pressures in this group. The following paragraphs will delve deeper into these results and discuss their implications.

**Table 8:** CS-ARDL estimation results.

	Variables	Long-run			Short-run		
		Coeff.	Z-stat	*P* value	Coeff.	Z-stat	*P* value
Full sample	lnGDP	− 0.3129***	− 9.02	0.000	− 0.376***	− 2.63	0.009
	lnNR	− 0.0185**	− 2.08	0.037	− 0.0236**	− 2.33	0.020
	lnREC	0.1009***	3.07	0.002	0.1766***	2.99	0.003
	lnEGL	− 0.27***	− 3.32	0.001	− 0.0592	− 0.66	0.512
	lnPOP	− 0.7934***	− 19.56	0.000	− 1.074	− 0.89	0.375
	lnRL	0.073**	2.29	0.022	0.068	1.59	0.112
	ECT (− 1)	− 0.3285***	− 7.97	0.000			
High income	lnGDP	0.3693**	2.49	0.013	0.8957*	1.89	0.058
	lnNR	− 0.2104***	− 2.84	0.004	− 0.0290	− 0.33	0.740
	lnREC	0.0959***	2.41	0.016	0.0050	0.10	0.922
	lnEGL	2.8381*	1.86	0.063	0.191	0.50	0.620
	lnPOP	− 0.67***	− 4.14	0.000	7.975	0.81	0.416
	lnRL	0.2783**	2.36	0.018	0.2035**	1.96	0.050
	ECT (− 1)	− 0.3494***	− 2.88	0.004			
Upper middle-income	lnGDP	− 0.6127***	− 7.02	0.000	− 0.4742**	− 2.35	0.019
	lnNR	− 0.0478***	− 4.20	0.000	− 0.0031	− 0.18	0.859
	lnREC	0.2675***	9.86	0.000	0.2061***	3.53	0.000
	lnEGL	− 0.2298***	− 3.39	0.001	− 0.0395	− 0.30	0.762
	lnPOP	− 0.0442	− 0.43	0.664	− 5.559*	− 1.87	0.062
	lnRL	0.0861***	2.69	0.007	0.0433	0.81	0.417
	ECT (− 1)	− 0.4764***	− 4.99	0.000			
Lower middle-income	lnGDP	− 0.7482***	− 8.06	0.000	− 0.4062**	− 2.32	0.020
	lnNR	− 0.0714***	− 3.53	0.000	− 0.0321***	− 3.79	0.000
	lnREC	0.4636***	7.71	0.000	0.2394**	1.88	0.060
	lnEGL	− 0.1746**	− 1.99	0.047	− 0.0164	− 0.25	0.803
	lnPOP	− 0.9409***	− 18.34	0.000	− 1.629	− 0.83	0.404
	lnRL	0.2862***	3.71	0.000	0.0171	0.45	0.653
	ECT (− 1)	− 0.287***	− 5.17	0.000			

***, **, and *signify significance at the 1%, 5%, and 10% levels, respectively.

In the full sample, economic growth exerts a statistically significant negative effect on the LCF in both the long and short run, suggesting that aggregate economic expansion continues to compromise environmental quality. This finding aligns with prior empirical research that documents the environmental costs of rapid growth, particularly in resource-driven and industrializing economies where environmental externalities are often inadequately internalized. However, disaggregating the results by income level reveals a nuanced picture of how economic growth interacts with environmental sustainability. In high-income countries, GDP is positively and significantly associated with LCF in both the short and long run, implying that economic growth enhances environmental quality in these nations. This pattern supports the Environmental Kuznets Curve hypothesis, which posits that as economies mature, the marginal environmental costs of growth diminish due to improvements in regulatory frameworks, advancements in green technologies, and stronger institutional capacities. In contrast, the relationship between GDP and LCF is significantly negative for both upper-middle-income and lower-middle-income countries across both time horizons. These results are consistent with recent findings by Refs.^15,18,42^, who report that economic expansion in developing contexts tends to exacerbate environmental degradation. The divergence across income groups can be attributed to structural economic differences within the OIC member states. In many lower-income and resource-rich economies, particularly those in SSA, economic activity is predominantly driven by natural capital exploitation, especially subsoil and agricultural resources. These economies often rely on energy-intensive production systems, suffer from weak environmental governance, and lack sufficient investment in clean technologies and green infrastructure. As a result, economic growth in these contexts tends to intensify ecological strain rather than alleviate it. Conversely, high-income OIC countries possess greater fiscal and institutional capacities that allow them to mitigate the adverse environmental impacts of growth. Through reinvestment in environmental sustainability measures such as renewable energy development, ecosystem restoration, and adoption of cleaner technologies, these countries are better positioned to decouple growth from environmental harm^31^. The contrasting outcomes across income groups underscore the importance of tailored policy responses. For developing economies, structural reforms aimed at diversifying economic activity, strengthening institutional quality, and investing in low-carbon technologies are essential for fostering a growth trajectory that is both economically inclusive and environmentally sustainable.

NR consistently exert a negative influence on LCF in the Full sample as well as across all income-based subgroups, with the effect being more pronounced in high- and lower-middle-income countries. This indicates that the dependence on natural resource extraction, particularly in fossil fuels and mineral exports, remains a critical threat to environmental sustainability within the OIC nations. While the short-run impact of NR is statistically insignificant in high- and upper-middle-income countries, possibly due to the presence of revenue stabilization mechanisms, diversified economies, or policy buffers, it is highly significant and negative in lower-middle-income countries. This suggests that in less-developed economies, the exploitation of natural resources leads to immediate environmental degradation, reflecting weaker environmental governance, lack of regulatory enforcement, and limited investment in green technologies. These findings are consistent with the conclusions of Sun et al.^34^ for G-11 nations, Zhou et al.^20^ for BRICS nation, and Zhang et al.^22^ focused on top resource-rich nations, all of which document the detrimental environmental consequences of intensified resource dependence. This result can be attributed to the fact that OIC nations possess a crucial portion of the world’s natural resources and are heavily dependent on them for their economies. Remarkably, their natural capital almost doubled from 1998 to 2018, indicating intensified resource exploitation. Furthermore, non-renewable subsoil rents constitute a major component of the GDP of these countries^31^. Therefore, the exploitation and extraction of these NR lead to water, air, and soil pollution, consequently increasing the EF and reducing the LCF. Additionally, the extraction of natural resources is an energy-intensive process that involves burning fossil fuels and depleting resources, which emit GHGs into the atmosphere, further reducing the LCF. This trend emphasizes the necessity for a more sustainable resource management strategy that balances economic expansion with environmental preservation.

Renewable energy consumption emerges as a robust determinant of environmental improvement across the Full sample and within all income-based subgroups of OIC nations. The positive and highly significant coefficients in both short-run and long-run estimates confirm the environmental benefits of transitioning to clean energy. Notably, the strongest effect of REC is observed in lower-middle-income countries, suggesting that these economies have the greatest potential to benefit from the expansion of renewable energy infrastructure. This finding underscores the argument that green energy development constitutes a critical policy tool for alleviating environmental stress, particularly in developing economies where reliance on fossil fuels remains high and regulatory capacity is often limited. The results imply that higher REC contributes to enhancing environmental changes in OIC nations. The findings highlight that promoting renewable energy sources can be an effective strategy for improving environmental outcomes in these countries. Our results align with those of Jahanger et al.^3^ on top ten SDGs nations, Wang et al.^45^ for Asian countries, and Aydin and Erdem^46^ for 18 EU nations. The favorable impacts of REC on LCF are because renewable energy sources emit fewer hydrocarbons. Consequently, increased utilization of renewable energy helps decrease carbon emissions, especially those generated by fossil fuel combustion^20^. The results indicate that OIC member countries should focus their policies on promoting renewable energy usage, as such a policy shift is beneficial for environmental improvement. REC helps increase the LCF by reducing ecological footprint and CO2 emissions. For example, Hydropower plants harness the energy generated by flowing water to generate electricity, making this process both clean and renewable. By utilizing the natural flow of water, hydropower provides an eco-friendly alternative to fossil fuels, lowering GHGs emissions and contributing to a more environmentally friendly energy landscape. Likewise, solar and wind energy systems generate electricity without causing air pollution. Biomass energy sources, including waste, wood, and biofuels, are capable of generating both electricity and heat, thus helping to minimize the volume of waste sent to landfills. Additionally, geothermal systems harness the Earth’s inherent heat to produce electricity, ensuring that no harmful emissions are emitted into the atmosphere^2^. Thus, it is imperative for OIC member states to prioritize renewable energy development within their national policy frameworks.

Globalization exhibits mixed effects on environmental sustainability across OIC countries. In the full sample and among upper- and lower-middle-income groups, the long-run impact of globalization is negative and statistically significant. This suggests that increased trade openness and foreign direct investment may aggravate environmental degradation in economies with weak environmental regulations. These results are consistence with those reported by Pata et al.^72^ for Latin American countries, Faizi et al.^73^ for OTS nations, Bektaş et al.^74^ for OECD countries, and Gaies et al.^26^ for MENA region, all of whom found that globalization tends to hinder environmental improvement in the absence of effective regulatory oversight. The channels through which globalization affects the environment appear to vary depending on countries’ income and institutional levels. In upper- and lower-middle-income OIC members, the results reflect the potential existence of the “pollution haven” hypothesis—Whereby pollution-intensive industries relocate from environmentally stringent high-income countries to those with laxer regulations. This underlines the urgent need for robust regulatory frameworks that address environmental externalities stemming from trade liberalization, FDI inflows, and industrial relocation in these income groups. Conversely, in high-income OIC countries, the long-run impact of globalization is positive and significant. This suggests that globalization may support the transfer of environmentally sound technologies and sustainable practices within more advanced institutional contexts. Such countries are better positioned to benefit from trade-induced environmental awareness, green technology adoption, and environmentally responsible FDI. These findings align with the existing literature emphasizing the role of globalization in promoting sustainable development, including Latif et al.^7^ for 48 Asian nations, Leal et al.^10^ for developed nations, and Erdoğan et al.^27^ for resource-rich SSA nations. Overall, these findings underscore the necessity for differentiated policy responses. While low- and middle-income countries must prioritize institutional strengthening to counteract the adverse environmental impacts of globalization, high-income countries should focus on leveraging globalization to promote sustainable development and green innovation.

Population growth shows a negative long-run effect on LCF, particularly in high-income and lower-middle-income OIC countries. This supports the view that growing populations increase environmental pressure by intensifying the demand for energy, water, land, and other ecological resources. These results in line with Aquilas et al.^43^ who observed that population growth adversely affects environmental sustainability. Interestingly, upper-middle-income countries exhibit an insignificant long-run relationship, indicating more complex or offsetting influences.

Lastly, the rule of law, a proxy for institutional quality, positively and significantly affects LCF in the long run across all income categories. This suggests that strong legal institutions and environmental governance mechanisms are critical in safeguarding ecological sustainability. These outcomes are in line with the findings of Hussain et al.^75^ for BRICS nations and Akpan et al.^76^ for 163 countries, which conclude that high institutional quality is instrumental in curbing environmental degradation, particularly by restraining fossil fuel dependence and enforcing environmental regulations. The short-run effects are weaker and mostly insignificant, indicating that the environmental benefits of institutional reforms accrue gradually over time. This finding is particularly relevant for lower-income OIC countries, where investments in institutional capacity-building and rule of law could yield substantial long-term ecological gains.

### Results of robustness check

The robustness check results presented in Table 9 provide further evidence supporting the main findings of the study. While the magnitude of the coefficients differs somewhat between the original estimation method and the alternative techniques (FMOLS, DOLS, ECM), the signs and overall significance levels remain consistent for most variables in the long-term findings. The FMOLS and DOLS techniques focus on long-run relationships. Across income groups and estimation methods, the long-run coefficients of REC and RL are consistently positive and significant, reinforcing their environmental benefits. NR and POP generally show a negative impact on LCF across all income groups and models. GDP is mostly negative and significant in full sample, upper- , and lower-middle-income groups, while it is positive in high-income countries, confirming the main results. EGL has a negative impact in the full sample and lower-income groups, while it is positive in high-income countries across all models (FMOLS, DOLS). The short-run ECM estimates further support these findings, with significant and negative ECT across all models, confirming stable long-run relationships. The direction and significance of most variables in ECM models align with the CS-ARDL outcomes, validating the robustness of the primary results.

**Table 9:** Robustness check results.

	Variables	Long-run results				Short-run results		
		FMOLS		DOLS		ECM		
		Coeff.	t-stat	Coeff.	t-stat	Variables	Coeff.	t-stat
Full sample	lnGDP	− 0.12***	− 41.14	− 0.49***	− 36.90	$\Delta$lnGDP	− 0.3245***	− 5.40
	lnNR	− 0.03***	− 25.69	− 0.17***	− 31.73	$\Delta$lnNR	− 0.0064	− 0.88
	lnREC	0.05***	50.32	0.86***	16.45	$\Delta$lnREC	0.09917***	5.37
	lnEGL	− 0.23***	− 9.69	0.56***	3.60	$\Delta$lnEGL	− 0.0072	− 0.15
	lnPOP	− 0.21***	− 46.37	0.19***	66.94	$\Delta$lnPOP	− 0.9551***	− 2.75
	lnRL	0.05***	13.68	0.29***	13.34	$\Delta$lnRL	− 0.0128*	− 1.74
						ECT (− 1)	− 0.2632***	− 11.59
High income	lnGDP	0.54***	5.70	1.76***	9.25	$\Delta$lnGDP	0.0699	0.15
	lnNR	− 0.01***	− 2.55	0.18***	7.93	$\Delta$lnNR	− 0.0038	− 0.06
	lnREC	− 0.10***	45.89	4.53***	5.34	$\Delta$lnREC	0.0381	0.30
	lnEGL	− 0.18***	10.45	0.06***	3.98	$\Delta$lnEGL	0.5254	1.05
	lnPOP	− 0.82***	− 36.72	− 1.62***	− 9.09	$\Delta$lnPOP	− 1.113*	− 1.80
	lnRL	0.17***	2.77	0.10***	11.90	$\Delta$lnRL	− 0.0268	− 0.16
						ECT (− 1)	− 0.2937***	− 4.02
Upper middle-income	lnGDP	− 0.22***	− 47.15	− 0.29***	− 15.76	$\Delta$lnGDP	− 0.4024***	− 4.53
	lnNR	− 0.01***	− 7.28	− 0.10***	− 2.26	$\Delta$lnNR	− 0.0029	− 0.27
	lnREC	0.26***	69.92	0.27***	10.56	$\Delta$lnREC	0.1287***	4.77
	lnEGL	− 0.36***	− 4.64	1.45***	5.24	$\Delta$lnEGL	0.0891	0.99
	lnPOP	0.026***	− 6.02	− 0.15***	− 26.05	$\Delta$lnPOP	− 1.143*	− 1.77
	lnRL	0.07***	24.12	0.79***	13.83	$\Delta$lnRL	− 0.0521***	–2.95
						ECT (− 1)	0.0725***	5.37
Lower middle-income	lnGDP	− 0.20***	− 18.34	− 0.64***	− 20.20	$\Delta$lnGDP	− 0.2714***	− 3.12
	lnNR	− 0.05***	− 29.32	− 0.09***	− 14.78	$\Delta$lnNR	− 0.0099	− 1.01
	lnREC	0.23***	5.51	0.94***	17.29	$\Delta$lnREC	0.2028***	3.21
	lnEGL	− 0.06***	− 4.44	− 0.29***	− 7.03	$\Delta$lnEGL	− 0.0416	− 0.87
	lnPOP	− 0.49***	− 44.02	− 4.81***	− 85.80	$\Delta$lnPOP	− 0.2685	− 0.38
	lnRL	0.01***	− 4.09	0.23***	5.08	$\Delta$lnRL	− 0.0020	− 0.30
						ECT (− 1)	− 0.2258***	− 7.95

***, **, and *signify significance at the 1%, 5%, and 10% levels, respectively.

Additionally, Table 10 presents the results of the Dumitrescu–Hurlin panel causality test, revealing bidirectional causal relationships between LCF and all explanatory variables. Specifically, GDP, NR, REC, EGL, POP, and RL each exhibit significant causality toward LCF (at the 1% or 5% significance levels), while LCF also significantly influences each of these variables in return. These findings highlight a dynamic interplay between environmental pressure and economic, demographic, institutional, and resource-related factors in OIC countries.

**Table 10:** Results of causality test.

Direction	Z-bar	*P* value	Causality	Result
GDP → LCF	10.8927***	0.0000	GDP → LCF	Bidirectional
LCF → GDP	6.1019***	0.0000	LCF → GDP	
NR → LCF	2.0080**	0.0446	NR → LCF	Bidirectional
LCF → NR	5.4405***	0.0000	LCF → NR	
REC → LCF	7.0195***	0.0000	REC → LCF	Bidirectional
LCF → REC	2.9535***	0.0031	LCF → REC	
EGL → LCF	2.9662***	0.0030	EGL → LCF	Bidirectional
LCF → EGL	7.2019***	0.0000	LCF → EGL	
POP → LCF	15.7340***	0.0000	POP → LCF	Bidirectional
LCF → POP	17.2264**	0.0000	LCF → POP	
RL → LCF	2.9411***	0.0033	RL → LCF	Bidirectional
LCF → RL	13.4594***	0.0000	LCF → RL	

* and **denote p value at 1% and 5%, respectively.

In particular, the causality from GDP to LCF lends support to the EKC hypothesis, which suggests that environmental degradation initially rises with economic growth but eventually declines as income levels increase. In the early stages of development, higher GDP often leads to intensified industrial activity, energy consumption, and resource use, thereby elevating the ecological footprint. However, as economies grow, they tend to adopt cleaner technologies, enforce environmental regulations, and transition toward less polluting sectors, leading to improvements in environmental quality. Conversely, the reverse causality from LCF to GDP implies that environmental degradation can negatively affect economic growth. A deteriorating environment may reduce labor productivity due to adverse health impacts, constrain agricultural and industrial outputs through resource depletion, and heighten vulnerability to climate-related risks. These challenges can hinder long-term economic development, especially in lower- and middle-income OIC countries with limited adaptive capacity.

## Conclusion and policy recommendations

Environmental issues have intensified in recent years, driven by climate change and global warming, presenting substantial risks to human well-being. OIC countries are affected by these issues, as highlighted by their average Environmental Performance Index (EPI) score of 35.7 in 2022 and a significant rise in GHGs emissions of 91% from 1990 to 2019 ^31^. This study examines how renewable energy, natural resources, role of law, economic performance, and economic globalization affect the LCF, a crucial measure of environmental improvement, across 36 OIC nations between 1996 and 2021. Importantly, the analysis was disaggregated by income level, into high-income, upper-middle-income, and lower-middle-income groups. This income-based stratification revealed substantial heterogeneity in the environmental effects of these variables. To achieve this, key econometric tests were applied to evaluate the characteristics of the data, confirming cointegration among the variables. The CS-ARDL methodology was then utilized to examine both short- and long-term impacts. The results demonstrate that economic expansion exerts a positive long-run effect on environmental quality in high-income OIC countries but is detrimental in upper- and lower-middle-income countries. Natural resources consistently undermine environmental sustainability across all income groups. Conversely, renewable energy consumption enhances environmental quality across all income groups. Globalization presents a dual picture: while it supports environmental improvement in high-income, it poses environmental risks in upper- and lower-middle-income. Population growth, across all income groups, further exacerbates environmental stress. The rule of law is shown to be a robust and consistent determinant of environmental quality across all income levels.

Based on the outcome above, the current study this study offers differentiated and targeted environmental policy recommendations for OIC countries, reflecting their diverse income levels and regional diversity. In light of the negative relationship between natural resource rents and environmental quality in high-income OIC nations, particularly hydrocarbon-dependent countries such as the GCC members, resource-rich economies must adopt stronger environmental oversight mechanisms. These countries should channel a portion of their resource revenues into sovereign green wealth funds to support ecological restoration, clean energy infrastructure, and climate adaptation. Coupled with their fiscal and institutional strength, they are well-positioned to pioneer environmental taxation on extractive industries and lead regional low-carbon transitions through investment in advanced renewable technologies, including green hydrogen and large-scale solar farms. In contrast, resource-rich but lower-income OIC members, particularly in Sub-Saharan Africa (SSA) and parts of Central Asia, face greater constraints in diversifying away from resource dependence due to fiscal pressures, weaker governance, and limited institutional capacity. For these nations, policy success hinges on access to international climate finance, technical assistance, and green technology transfer. Multilateral development banks and bilateral donors should offer concessional finance and capacity-building support to facilitate green industrialization and environmental policy implementation.

The positive and significant impact of renewable energy consumption (REC) on environmental quality across all income groups reinforces its critical role in sustainable development. High income economies such as GCC, with their robust financial and institutional capacity, are also well-positioned to scale up investments in renewable energy technologies, such as solar, wind, and green hydrogen. In upper-middle-income countries such as Türkiye, Malaysia, and several Central Asian and MENA OIC members, are encouraged to intensify their renewable energy transition by revising national energy targets, removing fossil fuel subsidies, and incentivizing clean energy deployment through mechanisms like feed-in tariffs, green bonds, and net metering. Regional cooperation in technology transfer and interconnection of power grids can be further developed across the MENA and Central Asian regions, capitalizing on shared resource potential and geographic proximity. For lower-middle-income OIC countries, particularly in SSA and South Asia, renewable energy policies should focus on increasing access to concessional finance from climate-related funds such as the Green Climate Fund (GCF), and on implementing decentralized solar and mini-grid systems for rural electrification. Policy tools like import tax waivers for renewable technology, community-based solar financing, and donor-backed energy access programs can play a crucial role in bridging the affordability and accessibility gap in these regions.

Economic globalization is observed to have mixed effects on environmental outcomes across OIC countries, with positive long-run benefits for high-income and negative effect for upper- lower-middle-income countries. Higher income countries should continue to integrate into global value chains by aligning industrial and trade policies with sustainability goals, ensuring that incoming FDI contributes to cleaner production. Strong governance, effective enforcement of environmental regulations, and adherence to international agreements are essential to maximize the environmental benefits of globalization. However, in upper- lower-middle-income OIC countries, globalization may exacerbate pollution due to weak regulatory frameworks and the risk of becoming pollution havens. Therefore, these countries must strengthen their environmental governance by enforcing pollution standards, adopting environmental impact assessment (EIA) in trade-related projects, and building monitoring systems. Aligning national development strategies with environmental clauses in international trade and investment agreements will help reduce the risks of environmentally harmful globalization.

Lastly, the role of institutional quality highlights that governance improvements significantly enhance environmental performance, across all income groups. OIC member governments, especially those with institutional weaknesses, should prioritize anti-corruption initiatives, digitization of environmental monitoring systems, and capacity building for environmental agencies. Local governments, civil society, and private sector actors must be actively engaged in environmental governance through inclusive partnerships. International partners, including Islamic financial institutions and development agencies, can support these efforts by providing technical assistance and financial support for regulatory modernization and the development of accountability frameworks.

### Limitations and future research

This study is subject to several limitations that warrant acknowledgment. First, the study does not incorporate important explanatory variables such as technological innovation, education, urbanization, or energy prices. The inclusion of these factors in future research could yield a more comprehensive and nuanced understanding of environmental sustainability in OIC countries. Second, the use of aggregate, country-level panel data limits the ability to explore subnational disparities and localized dynamics. Since environmental degradation, resource use, and policy responses can vary significantly within countries, future research should consider subnational or city-level analyses to identify localized patterns and policy needs. Third, although we stratified countries based on World Bank income thresholds to capture heterogeneity across income levels, we did not apply a formal threshold regression. Future research could employ endogenous threshold models to better capture nonlinearities in the environment–economy relationship. Lastly, employing advanced econometric techniques such as spatial models, panel quantile regressions, or mixed-method approaches, including case studies, to capture the heterogeneous effects and provide deeper contextual insights into environmental challenges and policy responses across different OIC regions.

## Supplementary Information

Supplementary Information.

## Data Availability

Data for GDP per capita, natural resources, renewable energy, and population were obtained from the World Development Indicators (WDI) database [https://databank.worldbank.org/source/world-development-indicators], and Economic globalization data were extracted from the KOF Globalization Index [https://kof.ethz.ch/prognosen-indikatoren/indikatoren/kof-globalisierungsindex.html#par_textimage_1585395273]. Data for load capacity factor extracted from the Global Footprint Network [https://data.footprintnetwork.org/#/countryTrends?cn=165&type=BCpc,EFCpc].
