# Supplementary material for: Evaluating the role of renewable energy natural resources and globalization in environmental quality in OIC countries
Source: Sci Rep. 2025 Sep 29;15:33496. doi: 10.1038/s41598-025-16872-y (PMC12480497; doi:10.1038/s41598-025-16872-y)
Supplement: Supplementary file 1 — Supplementary Information. [file 41598_2025_16872_MOESM1_ESM.docx]

**Supplementary Material**

**Appendices**

**Table S1:** Descriptive statistics

|  | Var | **N** | **Mean** | **SD** | **Min** | **Max** | **Skewness** | **Kurtosis** |
| --- | --- | --- | --- | --- | --- | --- | --- | --- |
| High-income | lnLCF | 104 | -1.85 | 0.80 | -2.84 | -.32 | .48 | 1.8 |
|  | lnGDP | 104 | 10.33 | 0.33 | 9.9 | 11.04 | .59 | 2.03 |
|  | lnNR | 104 | 3.16 | 0.37 | 2.27 | 4.01 | .07 | 2.81 |
|  | lnREC | 104 | -2.06 | 0.48 | -2.3 | .1 | 2.53 | 10.3 |
|  | lnEGL | 104 | 4.28 | 0.11 | 4.08 | 4.47 | .06 | 1.68 |
|  | lnPOP | 104 | 14.79 | 1.61 | 12.61 | 17.27 | .15 | 1.52 |
|  | lnRL | 104 | .81 | 0.12 | .4 | 1.01 | -.53 | 2.9 |
|  |  |  |  |  |  |  |  |  |
| Upper-middle-income | lnLCF | 390 | -.92 | 0.59 | -2.2 | .81 | -.23 | 2.74 |
|  | lnGDP | 390 | 8.12 | 0.66 | 6.43 | 9.51 | -.36 | 2.69 |
|  | lnNR | 390 | 1.65 | 1.62 | -3.15 | 4.18 | -.64 | 2.55 |
|  | lnREC | 390 | 1.59 | 1.48 | -1.61 | 4.03 | -.09 | 1.95 |
|  | lnEGL | 390 | 3.91 | 0.28 | 2.77 | 4.35 | -.81 | 3.93 |
|  | lnPOP | 390 | 16.98 | 1.12 | 14.85 | 19.29 | -.06 | 2.26 |
|  | lnRL | 390 | .11 | 0.67 | -3.82 | 1.01 | -2.21 | 10.25 |
|  |  |  |  |  |  |  |  |  |
| Lower-middle-income | lnLCF | 442 | -.01 | 0.77 | -1.25 | 3.14 | 1.77 | 6.91 |
|  | lnGDP | 442 | 6.99 | 0.68 | 5.91 | 9.1 | 1.11 | 4.2 |
|  | lnNR | 442 | 1.77 | 0.92 | -1.25 | 3.87 | -.48 | 3.24 |
|  | lnREC | 442 | 4.15 | 0.36 | 2.99 | 4.57 | -1.1 | 3.49 |
|  | lnEGL | 442 | 3.68 | 0.21 | 3.03 | 4.26 | -.63 | 3.74 |
|  | lnPOP | 442 | 16.52 | 1.41 | 13.96 | 19.44 | .46 | 2.59 |
|  | lnRL | 442 | .07 | 0.50 | -7.12 | .79 | -7.02 | 97.04 |

**Table S2:** Correlation matrix

|  | **Variables** | lnLCF | lnGDP | lnNR | lnREC | lnGL | lnPOP | lnRL |
| --- | --- | --- | --- | --- | --- | --- | --- | --- |
| High-income | lnLCF | 1.00 |  |  |  |  |  |  |
|  | lnGDP | 0.00 | 1.00 |  |  |  |  |  |
|  | lnNR | 0.34 | -0.25 | 1.00 |  |  |  |  |
|  | lnREC | -0.43 | 0.61 | -0.32 | 1.00 |  |  |  |
|  | lnEGL | -0.66 | 0.24 | -0.51 | 0.43 | 1.00 |  |  |
|  | lnPOP | -0.43 | -0.16 | 0.31 | 0.31 | -0.19 | 1.00 |  |
|  | lnRL | -0.03 | 0.52 | -0.54 | 0.38 | 0.48 | -0.45 | 1.00 |
|  |  |  |  |  |  |  |  |  |
| Upper-middle-income | lnLCF | 1.00 |  |  |  |  |  |  |
|  | lnGDP | -0.12 | 1.00 |  |  |  |  |  |
|  | lnNR | -0.07 | 0.11 | 1.00 |  |  |  |  |
|  | lnREC | 0.40 | -0.38 | -0.65 | 1.00 |  |  |  |
|  | lnEGL | 0.09 | 0.22 | -0.24 | 0.23 | 1.00 |  |  |
|  | lnPOP | -0.20 | 0.11 | 0.10 | -0.13 | -0.52 | 1.00 |  |
|  | lnRL | 0.12 | 0.22 | -0.52 | 0.34 | 0.36 | -0.05 | 1.00 |
|  |  |  |  |  |  |  |  |  |
| Lower-middle-income | lnLCF | 1.00 |  |  |  |  |  |  |
|  | lnGDP | 0.61 | 1.00 |  |  |  |  |  |
|  | lnNR | 0.50 | 0.31 | 1.00 |  |  |  |  |
|  | lnREC | 0.18 | -0.22 | 0.44 | 1.00 |  |  |  |
|  | lnEGL | 0.04 | 0.36 | 0.19 | -0.23 | 1.00 |  |  |
|  | lnPOP | -0.49 | 0.06 | -0.20 | -0.15 | 0.03 | 1.00 |  |
|  | lnRL | 0.10 | 0.07 | 0.11 | -0.11 | -0.01 | -0.09 | 1.00 |
